# Trabecular Attenuation of L1 in Adult Patients with Multiple Myeloma: An Observational Study on Low-Dose CT Images

**DOI:** 10.3390/hematolrep16040061

**Published:** 2024-10-17

**Authors:** Carlo Augusto Mallio, Valeria Tomarchio, Francesco Pulcini, Edoardo Verducci, Caterina Bernetti, Maria Antonietta Tafuri, Federico Greco, Luigi Rigacci, Bruno Beomonte Zobel, Ombretta Annibali

**Affiliations:** 1 Fondazione Policlinico Universitario Campus Bio-Medico, Via Alvaro del Portillo, 200, 00128 Roma, Italy; c.mallio@policlinicocampus.it (C.A.M.); v.tomarchio@policlinicocampus.it (V.T.); pulcini.francesco92@gmail.com (F.P.); e.verducci@policlinicocampus.it (E.V.); c.bernetti@policlinicocampus.it (C.B.); m.tafuri@policlinicocampus.it (M.A.T.); l.rigacci@policlinicocampus.it (L.R.); b.zobel@policlinicocampus.it (B.B.Z.); o.annibali@policlinicocampus.it (O.A.); 2 Research Unit Diagnostic Imaging, Fondazione Policlinico Universitario Campus Bio-Medico, Via Alvaro del Portillo, 200, 00128 Roma, Italy; 3 Research Unit of Hematology and Stem Cell Transplantation, Fondazione Policlinico Universitario Campus Bio-Medico, Via Alvaro del Portillo, 200, 00128 Roma, Italy; 4 Department of Radiology, Cittadella della Salute, Azienda Sanitaria Locale di Lecce, Piazza Filippo Bottazzi, 2, 73100 Lecce, Italy

**Keywords:** CT, low-dose, multiple myeloma, spine, MGUS

## Abstract

Background: The aim of this study was to evaluate the impact of trabecular attenuation of the L1 vertebral body in low-dose CT in adult patients with multiple myeloma (MM), smoldering multiple myeloma (SMM), and monoclonal gammopathy of undetermined significance (MGUS). Materials and Methods: The study population consisted of 22 patients with MGUS and 51 consecutive patients with newly diagnosed MM (SMM, *n* = 21; symptomatic MM, *n* = 36). CT scans were conducted using a 128-slice CT scanner (Somatom go.Top, Siemens, Munich, Germany). Low-dose whole-body CT scans were performed at a single time point for each patient. Trabecular bone density values were obtained by defining regions of interest on non-contrast images at the level of L1 vertebra. A threshold of *p* = 0.05 was applied to determine statistical significance. Results: The median Hounsfield unit (HU) value in patients with MGUS, SMM, and MM was 148 HU (range 81–190), 130 HU (range 93–193), and 92 HU (range 26–190), respectively, with a statistically significant difference between the groups (*p* = 0.0015). Patients with HU values ≤ 92 had lower progression-free survival with statistically significant differences compared to the group with HU values > 92 (*p* < 0.0499). Conclusions: This is the earliest evidence of the importance of evaluating L1 attenuation values in low-dose CT images in patients with MGUS, SMM, and MM. Further prospective studies could contribute to reinforcing these results and exploring the clinical applicability and generalization of L1 attenuation values in low-dose whole-body CT scans in routine clinical practice.

## 1. Introduction

Multiple myeloma (MM) is a malignant plasma cell neoplasm characterized by the infiltration of abnormal plasma cells in the bone marrow, leading to the overproduction of monoclonal immunoglobulins and a spectrum of skeletal complications [1,2]. The presence of increased osteoclast activity and the unbalance between resorption and formation, in association with an increased frequency of bone remodeling units, lead to rapid bone loss and the development of osteolytic bone lesions. Among these lesions, pathological fractures are frequent and contribute to the significant morbidity associated with MM [1,2,3,4,5].

In the spectrum of plasma cell disorders, there are precursor conditions such as monoclonal gammopathy of undetermined significance (MGUS) and smoldering MM (SMM) that might represent earlier stages of disease progression [1,2,5,6,7]. MGUS is characterized by the presence of a monoclonal protein (M protein) in the blood, without the typical clinical features of MM. SMM, on the other hand, represents an intermediate stage between MGUS and active MM, with higher levels of plasma cells and M protein, but still lacking the end-organ damage seen in active disease [1,2,5,6,7].

The identification and characterization of bone lesions is essential for the management and prognosis of MM patients, and imaging plays a crucial role in the diagnosis, staging, and follow-up of this context due to the excellent spatial and contrast resolution [8,9,10].

In recent years, low-dose computed tomography (CT) has emerged as a valuable imaging modality for the assessment of bone disease in MM patients, owing to its superior sensitivity for detecting subtle bone abnormalities compared to conventional radiography [3,4]. Lower-dose CT is considered a “morphological technique” with a high sensitivity for evaluation of even small osteolytic lesions and their characterization in terms of number, dimension, and internal density, which is fundamental for the diagnosis of MM considering the introduction of Slim-CRAB diagnostic criteria [11], in which the role of ^18^F-FDG PET/DWI-MRI as a more “functional technique” is also important to assess bone involvement.

While the presence of lytic lesions has traditionally been a focus of MM evaluation, attention has shifted towards understanding the changes in bone microarchitecture, particularly trabecular bone attenuation, as an important indicator of disease progression and fracture risk [12,13,14]. Trabecular bone attenuation, measured in Hounsfield units (HU), reflects the density and quality of trabecular bone and can provide insights into the extent of bone marrow infiltration and skeletal fragility in MM patients [12,13,14].

As the prevalence of MM and its precursor conditions continues to be a subject of clinical interest, the need for precise and non-invasive methods to assess bone health becomes increasingly vital.

Trabecular attenuation values of L1 on low-dose CT images have never been explored in adult patients with MGUS, SMM, and MM, and the potential importance of this metric in these patients is currently unknown.

This observational study aims to fill this knowledge gap by systematically evaluating trabecular attenuation of the L1 vertebral body in adult patients diagnosed with MM, including those with SMM and MGUS. We hypothesize that alterations in trabecular bone attenuation at this site are indicative of MM and may serve as a valuable biomarker for discriminating between SMM and MGUS. To achieve this goal, we will employ low-dose CT imaging, which has demonstrated superior sensitivity and specificity in detecting early bone changes in MM patients.

## 2. Materials and Methods

### 2.1. Study Design

This retrospective observational study was conducted at a single center, adhering to the principles outlined in the Declaration of Helsinki. Ethical approval was granted by the Ethical Committee (protocol: 98.21 OSS), and all participating patients provided informed consent for the utilization of their clinical and imaging data for research purposes. The ethical approval and the date of obtaining consent of the ethics committee were duly recorded in accordance with the study’s compliance protocols.

### 2.2. Patient Selection

The study cohort consisted of adult consecutive patients recruited at the Unit of Hematology and Stem Cell Transplantation of our University Hospital between June 2017 and March 2023. Patients were divided into the three following groups:

- Patients with diagnosis of MGUS;
- Patients with diagnosis of SMM;
- Patients with diagnosis of MM.

To qualify for inclusion, patients had to meet the following criteria: they should not have received any systemic therapy, and they should have undergone a low-dose whole-body CT scan. For patients receiving more than one low-dose whole-body CT scan we considered for the analysis the first imaging available. Patients with bone disorders or those who had previously received one or more doses of bisphosphonates were not included. Likewise, bone lesions that had been treated with antalgic radiotherapy were not taken into account.

### 2.3. Hematologic Assessment

The study population consisted of 22 patients with MGUS and 51 consecutive patients with newly diagnosed MM (SMM, n = 21; symptomatic MM, n = 36).

Diagnosis of MGUS, SMM, and symptomatic MM was made according to the International Myeloma Working Group (IMWG) criteria and its updated version [11,15]. Patients were informed about the benefits and potential risks of CT and underwent it parallel to the MGUS–myeloma work-up. Laboratory work-up included blood count and chemistry (e.g., creatinine and calcium evaluation), serum and urine protein electrophorese and immunofixation electrophoresis, quantification of serum immunoglobulin (Ig) and free light chain (κ, λ, and κ/λ ratio), 24 h urinary protein excretion, and serum β2-microglobulin and lactate dehydrogenase levels. Bone marrow aspiration was performed in all MGUS patients, while bone marrow aspiration and biopsy were performed in all MM patients.

No patient received bisphosphonates, or chemotherapy or radiation therapy before CT was performed. Furthermore, some patients presented symptoms or signs of infection, inflammation, or uncontrolled hyperglycemia, or received a steroid within a few days before the exam.

Patients with symptomatic MM were evaluated at diagnosis, considering prognostic factors such as ISS (International Staging System) [16] and were treated with daratumumab or non-daratumumab combined chemotherapy in the frontline setting. They were evaluated for response according to IMWG criteria [17].

### 2.4. CT Imaging

CT scans were conducted using a 128-slice CT scanner (Somatom go.Top, Siemens, Germany). Low-dose whole-body CT scans were performed at the first diagnosis for each patient.

The low-dose whole-body protocol was adjusted as follows: (kV = 120, mAs = 40, pitch = 1.2 mm, slice thickness = 3 mm). Mean dose length product = 676 mGy x cm; mean computed tomography dose index = 5.7 mGy. Scans were obtained without administration of an iodine-based contrast agent.

**Figure 1:**
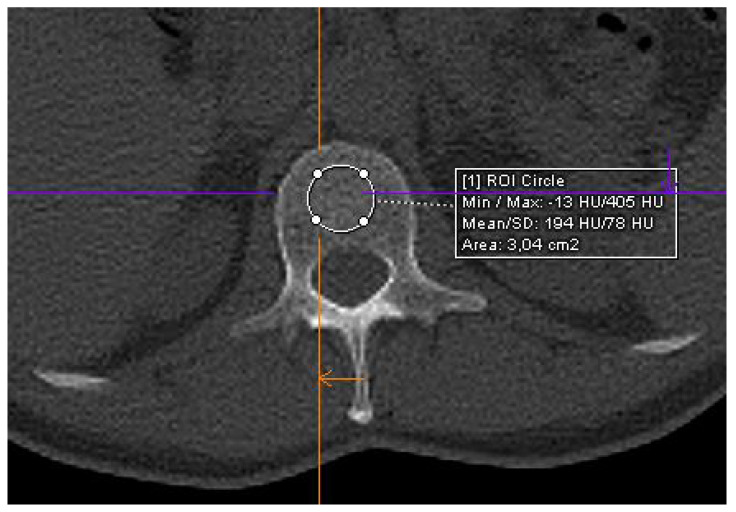
Axial CT image showing ROI placement at the level of the trabecular bone of the L1 vertebra. Purple and orange lines are for anatomical reference of correct positioning.

Image analysis was conducted on a dedicated workstation, utilizing the appropriate window settings for bone assessment (width: 2500 HU; window level: 480 HU) [18]. Trabecular bone density values were obtained by defining regions of interest (ROIs) on non-contrast images at the level of L1 vertebra (Figure 1). We chose the L1 vertebra because it has been shown by previous studies to be the best vertebra to take for this type of analysis [12,13,14]. We focus on L1 vertebral measurements in this study for several key reasons. First, outcomes at L1 are as accurate, if not more so, than those obtained at other vertebral levels, including multilevel evaluations. The L1 vertebra is easily recognizable, enhancing both efficiency and reproducibility. It is also routinely captured in standard chest and abdominal CT scans, significantly increasing its potential for broader screening applications [12,13,14].

ROIs were placed at the level of a normal-appearing trabecular bone. If osteolytic bone lesions or negative values, likely indicative of vertebral hemangioma with a high fatty bone marrow content, were detected at the level of an L1 trabecular bone, alternative ROIs were drawn at the level of the adjacent vertebral trabecular bone. Attenuation measurements were performed at the level of the L1 bone for all the patients, except for 1 patient in which the ROI was placed at D12, 9 patients in which measurements were taken at L2, 2 patients in which the L3 vertebra was sampled, and 2 patients who were evaluated at L4. The differences in vertebral level selected for the analysis were chosen to avoid vertebral lesions of the trabecular bone and/or spinal fractures. Cortical measurements were not included in the analysis.

At each measurement, a circular ROI was delineated by a consensus between one radiologist (C.A.M., 12 years of experience) and one resident in radiology (F.P., 4 years of experience).

### 2.5. Statistical Analysis

Baseline characteristics were analyzed for significance of differences between groups by one-way analysis of variance for continuous variables and chi-squared test for categorical variables. Patients with symptomatic MM were categorized into high and low HU levels using the median value as a cut off. Survival was assessed with a prospective follow-up. Overall survival (OS) was defined from the date of diagnosis until death from any cause; progression-free survival (PFS) was defined from the date of diagnosis until progression or death from any cause, and survivors were anonymized at the time of last contact. The associations of the HU value with OS and PFS were analyzed using Kaplan–Meier curves and compared using the log-rank test. A threshold of *p* = 0.05 was applied to determine statistical significance.

## 3. Results

### 3.1. Clinical Data of the Patients

Among the 22 patients with MGUS, the median age was 62 years old, and they presented with essentially normal parameters and with a median M-protein of 0.5 g/dl; in 87% (19/22) of cases, this was IgG, and the median value of plasma cells in the bone marrow was 4%. In the SMM group, the median age was 67 years old, the median MC value was 1.9 g/dl, which was IgG in most of the cases (17/21, 81%), and they presented a median involvement of plasma cells in the bone marrow of 15% (Table 1).

In the MM group, we observed an increase in M-protein, the percentage of plasma cells in bone marrow, K/L ratio, Beta2microglobulin. As shown in Table 1, the median age of the MM group was 70 years old, 20/36 (55%) of which were male and 16/36 (45%) female, M-protein was found in 42% (15/36) of cases to be non-IgG, and urinary immunofixation was positive for Bence Jones in 47% (17/36) of cases.

According to some of the most important prognostic factors such as the level of hemoglobin, K/L ratio, quantity of M-protein, and % of bone marrow infiltration by plasma cells, we found a difference that was statistically significant between the three groups; age was similar between groups.

Among the 36 patients with MM, 6 (17%) had ISS I, 18 (50%) had ISS 2, and 12 (33%) had ISS 3; 47% (17) of patients started daratumumab-combined chemotherapy as an induction in first-line treatment. Responses to treatment according to the IMWG were complete remission (CR) and very good partial Response (VGPR) in 64% (23/36) of cases.

**Figure 2:**
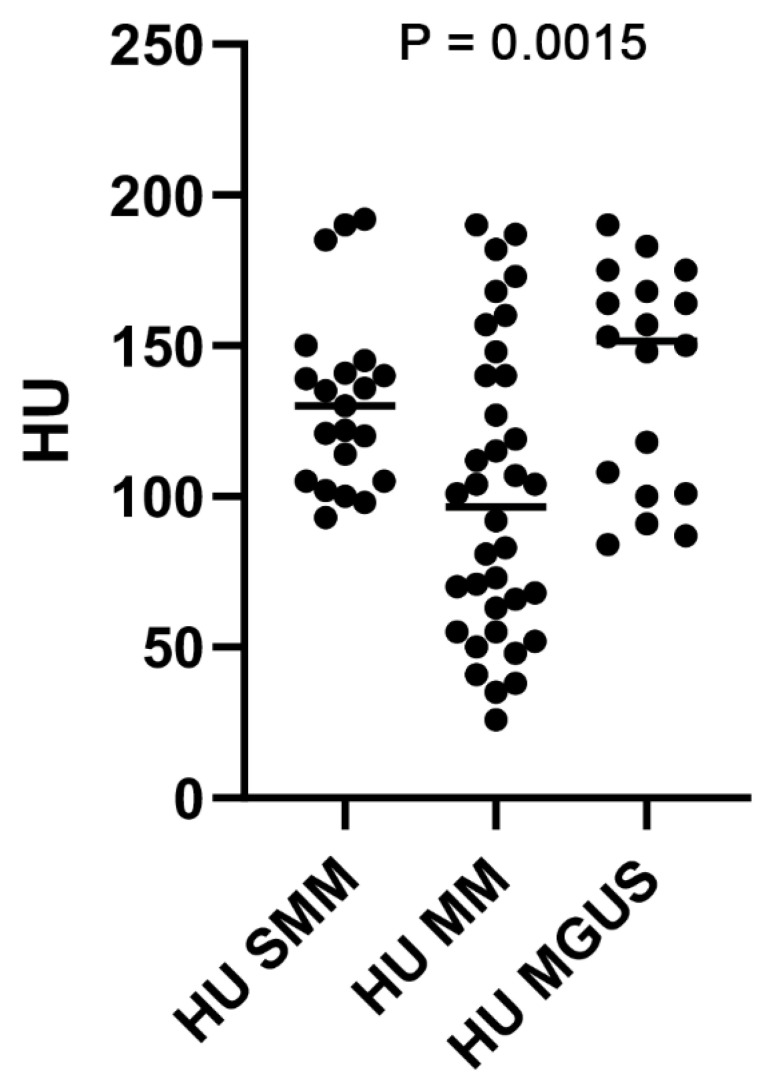
Box plot of HU value in patients with MGUS, smoldering MM, and symptomatic MM. ANOVA, analysis of variance.

Figure 2 shows box plots of the HU value in patients with MGUS, SMM, and symptomatic MM. The median HU values in patients with MGUS, SMM, and symptomatic MM were 148 HU (81–190), 130 HU (93–193), and 92 HU (26–190), respectively, with a difference that was statistically significant between groups (*p* = 0.0015 by analysis of variance).

### 3.2. Distribution of Patient Characteristics According to HU

Patients with MM were divided into two groups according to the HU value: 18 had an HU value ≤ 92 and 18 > 92. Table 2. The clinical background of abnormal HU values was evaluated by comparing the two groups with regard to M-protein levels, ISS, K/L ratio, presence of Bence Jones, percentage of plasma cell infiltration in bone marrow evaluated by biopsy, clinical responses, and the presence of more than three osteolytic lesions.

Patients with HU values ≤ 92 presented with a more aggressive disease: 8/18 (45%) had ISS 3 at diagnosis vs. 4 (22%) in the other group; 13/18 (72%) patients with a HU value ≤ 92 had a value of infiltration of plasma cells in the bone marrow > 40% (median value of MM group) vs. 9/18 (50%) in the other cohort. The group with an HU value ≤ 92 showed more cases with a high K/L ratio (10/18, 55% vs. 4/18, 22%) (considering median value of the MM group of 19.5), a low percentage of positive responses as CR and VPGR (10/18, 55% vs. 13/18, 72%) and a higher level of M-protein (12/18, 67% vs. 7/18, 39% ≥ 2.1 g/dL).

These trends observed in the HU ≤ 92 group were not statistically significant. Finally, the group with HU values ≤ 92 presented more than three osteolytic lesions upon whole-body CT in 12/18 (67%) vs. 6/18 (33%) cases (*p* = 0.0455). See Table 2 for the full list of results.

### 3.3. Correlations between HU and Clinical Parameters in MM

Comparing linear variable of HU values with important prognostic data such as M-protein, percentage of plasma cells in bone marrow, hemoglobin, and K/L ratio, we found a negative correlation between HU value and M protein, percentage of plasma cells in bone marrow, and K/L ratio, with an R value of R −0.1457, R −0.0234, and R −0.05098, respectively, with no statistically significant difference but with a clinically correct trend. A concordant positive trend between HU and Hb values (R 0.05856), was also related to the disease tumor burden in this case: higher tumor burden, lower HU values.

### 3.4. Survival Analysis

**Figure 3:**
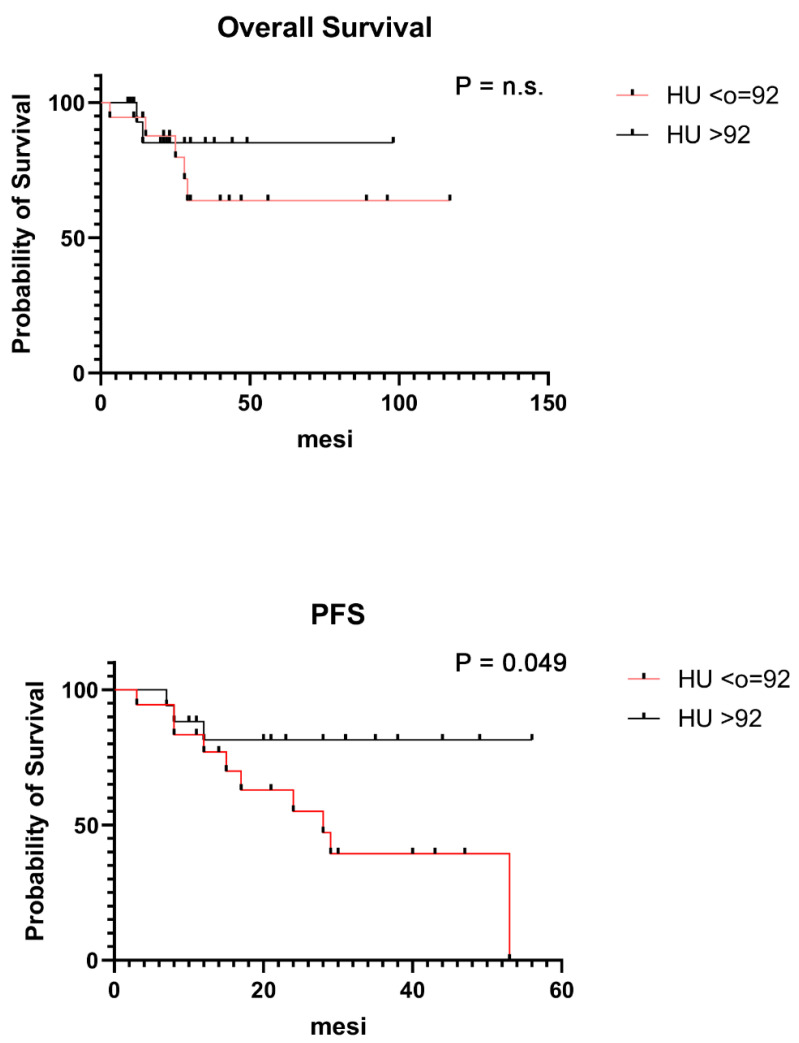
Kaplan–Meier curves showing the survival analysis of MM patients divided according to HU values.

Seven patients (25%) died over a median follow-up of 25 months (3–117); in particular, four died due to progressive disease and three due to other causes (infective complications). Five (71%) out of seven patients died in the group with a lower HU value. The Kaplan–Meier curves for OS and PFS of both groups are shown in Figure 3. Patients with HU values ≤ 92 showed a lower survival rate than those without such lesions, but the difference did not reach statistical significance (*p* = 0.4208); the median OS was reached for HU > 92 group (44 months). The 2-year OS rate was 85% for both groups. Moreover, patients with HU values ≤ 92 showed a lower PFS with a statistically significant difference compared with those in the group with HU values > 92. Median PFS was not reached in the second group whereas that of 28 months was reached in the first, and the PFS 2-year rates were 80% vs. 40%, respectively (*p* < 0.0499).

## 4. Discussion

**Figure 4:**
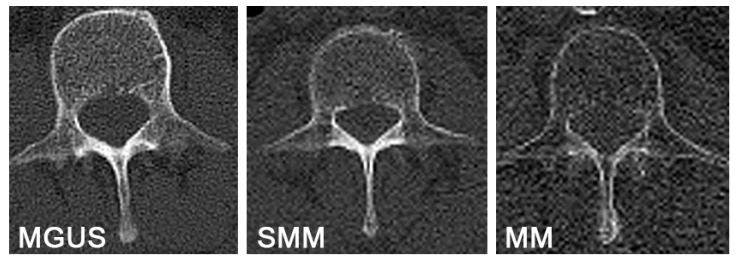
Axial low-dose CT images at the level of L1, showing the progressive reduction in bone density across the three groups: monoclonal gammopathy of undetermined significance (MGUS), smoldering multiple myeloma (SMM), and multiple myeloma (MM).

In the present study, we found that the values of L1 attenuation upon a low-dose whole-body CT can significantly distinguish MGUS, SMM, and MM. Moreover, patients with MM showed statistically significant lower HU values with respect to MGUS and SMM together. Remarkably, lower attenuation values in our sample were associated with worse PFS in MM. These results and correlations are the earliest evidence on the importance of assessing attenuation values of L1 upon low-dose whole-body CT imaging in patients with MGUS, SMM, and MM (Figure 4).

Normative population-based (>20,000 adults) values of L1 trabecular attenuation were reported in a previous study from images acquired with 120 kV [12], while the clinical importance of this opportunistic approach has been underlined in other studies [19,20]. For instance, Graffy et al. reported that the prevalence of moderate or severe vertebral fractures was 32.5% with L1 attenuation ≤ 90 HU and increased up to 49.2% with L1 attenuation ≤ 50 HU [20].

It should be noted that here we used a low-dose CT protocol, and the technical settings have a profound impact on trabecular bone as compared with soft tissues [21]; therefore, the results we report in the present study should not be generalized to full-dose images or to significantly different technical protocols.

To the best of our knowledge, this is the first study to apply this opportunistic approach to low-dose CT images in MM.

The ability to differentiate between MGUS, SMM, and MM is a crucial aspect of clinical decision-making, as the management of these conditions differs significantly between settings [22,23,24].

Considering the actual diagnostic guidelines [11] in the work-up of a patient with MM is necessary: either lower-dose CT as a morphologic technique or ^18^F-FDG PET/DWI-MRI as a functional technique can be used in order to detect lytic lesions; regardless, this goal is sometimes challenging due to the dimensions or the density of lesions.

Agreement between lower-dose CT and ^18^F-FDG PET is reported to be higher than 85% [25], while concordance between lower-dose CT and MRI is roughly 70% [26]; moreover, PET cannot capture negative-HU lytic lesions, which can also be missed by DWI-MRI as demonstrated in the study by Zambello et al. [27]. In this study, the researchers investigated two types of abnormal lesion through CT, one with positive and the others with negative HU values in 18 MM patients. The histology of negative-HU lesions showed infiltration by neoplastic plasma cells scattered among adipocytes, although these types of lesions did not appear upon ^18^F-FDG PET/DWI-MRI imaging. According to these results, lower-dose CT adds specific information to distinguish between MGUS vs. SMM vs. MM more precisely, and with the application of HU evaluations, it could also assume the role of a “functional” exam and add information that could be missed by PET or MRI.

The L1 attenuation values obtained from low-dose whole-body CT scans provide a non-invasive and readily available parameter that can contribute to this differentiation. The lower attenuation values observed in patients with MM may reflect the higher tumor burden and bone marrow involvement in this advanced malignancy.

Koutoulidis et al. enrolled a group of 76 patients with myeloma who had undergone whole-body low-dose CT to investigate the pattern of appendicular bone medullary attenuation [28]. They reported that bone medullary attenuation differed significantly among mixed, nodular, and diffuse CT-based appendicular medullary cavity patterns in the femurs and humeri, suggesting that peripheral medullary patterns of attenuation on whole-body low-dose CT might distinguish patients with MM from those with diffuse marrow involvement.

More recently, Gu et al. applied-dual energy CT calcium-subtracted attenuation to assess bone marrow infiltration in patients with MM [29]. The authors found a statistically significant correlation between calcium-subtracted attenuation and bone marrow plasma cell infiltration percentage (Spearman’s rho: + 0.79, *p* < 0.001), suggesting this as an objective measure of marrow involvement that is potentially helpful for the earlier detection of disease. In the present study, we used a different approach because we quantified the trabecular bone without any calcium subtraction on low-dose CT images; thus, our results are more likely to reflect bone mineral loss in MM.

Moreover, the study by Zijlstra et al. [30] analyzed 1605 vertebrae of 143 patients with MM by CT scan, demonstrating that a lower HU score, indicating lower bone marrow density, was correlated with higher fracture risk.

In our study, lower HU values correlated in a statistically significant way with the presence of more than three lytic lesions, demonstrating that these groups of patients were at a higher fracture risk and that the clinicians could adopt, for example, more frequent bone monitoring or preventive bone strategies, such as vitamin D replacement, biphosphate administration, use of orthopedic devices, or radiotherapy in a localized area, for these patients.

Our findings on the association between lower attenuation values and worse progression-free survival suggest the potential utility of L1 attenuation as a prognostic marker. This finding is of clinical relevance, as predicting the progression of MGUS and SM to MM is a matter of great interest for timely intervention [22,23,24].

The differentiation between MGUS, SMM, and MM is a fundamental challenge in the clinical management of plasma cell disorders. Our study investigated the potential of L1 attenuation values derived from low-dose whole-body CT scans as a tool for the differentiation, prognostication, and individuation of MM patients who are more fragile from a bone point of view. The lower L1 attenuation values observed in the MM patients in comparison to the MGUS and SMM patients are of particular interest. This disparity likely reflects the increased tumor burden and bone marrow involvement characterizing MM. This study underscores the effectiveness of low-dose CT in assessing trabecular attenuation in the L1 vertebral body, providing valuable insights into bone health for patients diagnosed with MM, SMM, and MGUS. One significant advantage of low-dose CT is its capability to evaluate bone fragility. Early detection through this imaging technique allows for timely interventions, which can substantially reduce the risk of fractures and ultimately enhance patient outcomes. Furthermore, low-dose CT offers a non-invasive method for monitoring disease progression and evaluating treatment efficacy over time. The ability to identify bone changes early and implement personalized treatment strategies contributes to improved quality of life for these patients. Additionally, low-dose CT enables the evaluation of all skeletal segments while reducing radiation exposure compared to that in standard CT scans. For these categories of patients, who often require frequent imaging assessments throughout their treatment, minimizing radiation risk is crucial. By incorporating low-dose CT into clinical practice, healthcare providers can effectively monitor bone health and disease progression without exposing patients to unnecessary radiation.

Limitations of the present study should be considered when interpreting the results: First, the retrospective nature of it limited the availability of clinical information and the generalizability of results. Second, the relatively low number of patients in each group characterizes this study as exploratory. Further studies with a larger patient population are needed to explore these findings in greater detail. Third, CT trabecular attenuation is a spurious measure that is impacted by many features, including bone mineral density and balance between red and yellow bone marrow, so it is not a direct measure of MM cell infiltration. However, with the opportunistic approach used in the present paper, we could obtain clinically relevant information with a single and fast measure in three homogeneous groups of patients (i.e., MGUS, SMM, and MM).

Further prospective investigations are warranted to validate these findings across a broader patient population and to explore the practical implementation of L1 attenuation values in routine clinical practice. Such efforts could lead to enhanced diagnostic precision and a deeper understanding of disease progression or become a marker of bone repair in the case of patients responsive to treatment, ultimately improving the management of patients along the MM spectrum.

## 5. Conclusions

In conclusion, our study presents the earliest evidence of the importance of assessing L1 attenuation values upon low-dose whole-body CT imaging in patients with MGUS, SMM, and MM. These values show significant discriminatory power between these conditions and offer potential prognostic insight, indicating their potential role in enhancing the clinical management of patients along the multiple myeloma spectrum. Further prospective studies would be helpful to strengthen these findings and explore the clinical applicability and generalizability of L1 attenuation values in low-dose CT in routine patient care.

## Figures and Tables

**Table 1:** Characteristics of the different groups.

	MGUS n° pt 22 N (%)	SMM n° pt 21 N (%)	MM n° pt 36 N (%)	*p*
Male/Female (%)	11 (50%)/11 (50%)	7 (33%)/14 (67%)	20 (55%)/16 (45%)	
Median age (min-max)	62 (39–82)	67 (37–85)	70 (50–91)	NS
HU value (min-max)	148 (81–190)	130 (93–192)	92 (26–190)	
Hb (g/dL) (min-max)	14 (10.6–17)	12.9 (10.8–15.8)	11.7 (6.7–16)	0.004
Creatinine (g/dL) (min-max)	0.8 (0.6–1.85)	0.9 (0.65–1.4)	0.9 (0.5–3)	
Calcium (g/dL) (min-max)	9 (8.7–10)	9 (8.6–10.3)	9.4 (8.2–18)	
Albumin (g/dL) (min-max)	4 (2.5–4.4)	4 (2.4–4.8)	3.8 (3–4.8)	
K/L chain ratio (min-max)	1.8 (0.8–13)	3 (0.8–36)	19.5 (1–1200)	0.002
LDH (U/L) (min-max)	186 (134–490)	185 (107–302)	195 (119–300)	
B2microglobulin (mg/dL) (min-max)	1.6 (1–3)	2.05 (1–6)	4 (2–19)	
M-protein g/dL (min-max)	0.5 (0.2–1.5)	1.9 (0.7–2.9)	2.1 (0.1–5)	0.023
IgG/non IgG	19(86%)/3 (14%)	17 (81%)/4 (19%)	21 (58%)/15 (42%)	
BJ pos/neg	9 (41%)/13 (59%)	5 (24%)/16 (76%)	17 (47%)/19 (53%)	
Bone marrow—plasma cells (%) (min-max)	4 (1–10)	15 (40–10)	40 (5–80)	<0.0001
ISS I/II/III			6(17%)/18 (50%)/12 (33%)	
Daratumumab therapy-based vs. not			17(47%) vs. 19 (53%)	
CR + VGPR			23 (64%)	

Abbreviations: BJ: Bence Jones; ISS: International Staging System; CR: complete remission; HU: Hounsfield units; IgG: immunoglobulin G; LDH: lactic dehydrogenase; MGUS: monoclonal gammopathy of undetermined significance; MM: multiple myeloma; SMM: smoldering multiple myeloma; VGPR: very good partial response.

**Table 2:** Characteristics of symptomatic MM patients classified according to HU value.

	HU ≤ 92 (N° pt 18) N (%)	HU > 92 (N° pt 18) N (%)	*p*
Stage			NS
ISS III	8 (45%)	4 (22%)	
ISS I/II	10 (55%)	14 (78%)	
M-protein			0.069
≥2.1 g/dL	12 (67%)	7 (39%)	
<2.1	6 (33%)	11 (61%)	
Bone marrow—plasma cells (%)			NS
≥40%	13 (72%)	9 (50%)	
<40%	5 (28%)	9 (50%)	
K/L ratio			NS
≥19.5	10 (55%)	4 (22%)	
<1.6	8 (45%)	14 (78%)	
BJ			NS
Pos.	8 (45%)	9 (50%)	
Neg.	10 (55%)	9 (50%)	
Response			NS
CR + VGPR	10 (55%)	13 (72%)	
PR o SD	8 (45%)	5 (28%)	
Type of MC			NS
non IgG vs.	8 (45%)	7 (39%)	
IgG	10 (55%)	11 (61%)	
Osteolithic lesions			0.045
>3	12 (67%)	6 (33%)	
<3	6 (33%)	12 (67%)	

Abbreviations: BJ: Bence Jones; ISS: International Staging System; CR: complete remission; HU: Hounsfield units; IgG: immunoglobulin G; MM: multiple myeloma; VGPR: very good partial response.

## Data Availability

The dataset used and/or analyzed during the current study is available from the corresponding author on reasonable request.
